# Book Reviews

**Published:** 1813-02

**Authors:** 


### CRITICAL ANALYSIS
### OF RECENT PUBLICATIONS
IN THE
### DIFFERENT BRANCHES OF PHYSIC, SURGERY, AND MEDICAL PHILOSOPHY.

*The Morbid Anatomy of the Liver; being an Inquiry into the Anatomical Character, Symptoms, and Treatment, of certain Diseases which impair or destroy the Structure of that Viscus. Order I. Tumors. Part I. On the Tubera Circumscripta, and Tubera Diffusa. By* J. R. Farre, M.D. Imperial 4to.; pp. 22; plates. Longman and Co. 1812.

FROM the plan which the author has adopted of publishing his work *in fasciculi*, we have no intimation of the extent to which it may be carried, nor of the period when it may terminate. The importance of the subject, however, claims our immediate attention, and we shall forthwith proceed to acquaint our readers with what is contained in the present specimen, which, in our opinion, gives promise of a great and standard performance. We shall not stop to quibble about the term *morbid anatomy*, nor attempt a vapid peroration on the importance and utility of this interesting branch of our science, the study of which hitherto has been much neglected and injudiciously pursued. The physician consoles himself that the disease of which, on dissection, he contemplates, the ravages was incurable; the surgeon is intent on enriching his collection with another portion of deranged structure; but neither are sufficiently diligent in ascertaining all the intermediate changes in the progress, from the healthy to the diseased and irrecoverable condition of the organ. It does not appear to us that Dr. Farre, in this respect, has advanced beyond his predecessors; but, from his strong and clear power of observing, his freedom from prejudice, united to a sound and steady judgment, we augur that in the course of his laborious undertaking he will achieve something more than a dry detail of facts.

He commences with the following definitions, which we transcribe for the right understanding of the work.

"Tumors.—Swellings, either circumscribed or diffused, generally differing in structure from the natural textures of the affected organs, and increasing in bulk by an inherent growth.

"Tubera.—Tumors of a cellular structure and fungous nature, producing, in general, remarkable elevations on the surfaces of the affected parts.

"Tubera

"TUBERA CIRCUMSCRIPTA.—Tubera determinate in their figure, and limited in their seat chiefly to the lungs.

"TUBERA DIFFUSA.—Tubera indeterminate in their figure, diffused through the affected organ, or dispersed in many textures of the body."

In his definition of Tubera Circumscripta, Dr. Farre has corrected Dr. Baillie, who describes the disease as "*the large white tubercle of the liver.*" Dr. F. contends that "the epithets large, white, are not characteristic, being common to both species, and belong indeed in a more remarkable degree to Species II. It seemed to him also objectionable to apply the term Tubercles indifferently to these large tumors, to certain irregularities of surface produced by chronic inflammation, and to scrofula."

Of the first order, Tumores, the liver is chiefly subject to Tubera Circumscripta, thus described by Dr. Farre:

"Their color inclines to a yellowish white; they elevate the peritoneal tunic of the liver; and their projecting surfaces, slightly variegated with red vessels, deviate from a regular swell by a peculiar indentation at or near their centres, which are perfectly white and opaque. They vary much in size, which depends on the duration of each tuber; for at its first appearance it is very minute, but during its growth it assumes the character above described, and at its maturity exceeds an inch in its diameter. They adhere intimately to the liver, and their figure is well defined. In the interstices of the tubera, the liver is paler and more flabby, its cohesion is weaker than natural, and slight effusions of blood are sometimes found. They commonly remain distinct at the surface of the liver, but internally they ultimately coalesce, and form immense morbid masses which pervade its substance. The patient often lives until the mass occupies the greatest part of the abdomen, and the natural structure of the liver is nearly supplanted. They possess so close a cellular structure, that the section of them, at first view, appears solid and inorganic; but on the edge of the knife, by which they have been dissevered, an opaque white fluid, of the consistence of cream, is left, and a fresh portion of this fluid is gathered on it at each time that it is repassed over the surface of the section. Their cellular structure becomes more apparent after long maceration.

"SYMPTOMS.—The patient suffers pain in the region of the liver, languor, loss of appetite, and cough; but, until the liver, by the growth of the tubera, descends below the hypochondria, a distinct judgment of the case cannot be formed: then the functions of the alimentary canal are more impaired, the body wastes, and the enlargement of the liver, its hardness and remarkable irregularity of surface, may be distinguished through the parietes of the abdomen. In the advanced stage the patient is distressed by its enormous bulk; the respiration is oppressed; the bowels are prone to diarrhœa. Neither jaundice nor serous effusion into the peritoneum are symptomatic of this disease: they may be conjoined, but it is an accidental circumstance, rather than a necessary consequence."

Two well-related cases, with the dissections, and illustrated by a plate, remarkable for the beauty of its execution and the accuracy of its delineation, terminate the author's history of Tubera Circumscripta; or, as the disease has been named by Dr. Baillie, "the large white tubercle of the liver." Besides his objection to the term adopted by this distinguished writer, Dr. Farre dissents from his notion that the disease is scrofulous, and grounds his opinion on these circumstances:

"First, the Tubera Circumscripta are distinctly allied to the Tubera Diffusa, which unquestionably fall under the tribe of fungous diseases. Secondly, the Tubera Circumscripta differ from the Tubercula Strumosa in their character and termination."

Dr. Farre admits that the disease is occasionally "conjoined" with scrofula; and, should the reasons that he has offered for rejecting the notion of Dr. Baillie be deemed slight or insufficient, will a candid objector regard that celebrated physician's opinion to be founded on strong evidence, when, describing the nature of the large white tubercle of the liver, he says, "It resembles more the ordinary appearance of scirrhus in other parts of the body. In one or two instances of it, however, I have observed a thick sort of pus, resembling very much the pus from a scrofulous sore; and, *therefore*, I am rather disposed to think that this tubercle may be of a scrofulous nature." Perhaps we have erred in stating this difference of opinion, because neither of the authors in question speak positively on the subject; but, when Dr. Farre is "inclined to believe," and Dr. Baillie "rather disposed to think," we know that the cautiously-worded sentence is received by the many as law.

"II. Tubera Diffusa.—Character.—These tumors not only pervade the substance of the liver in a distinct or in a confluent form, but also appear at its surface, elevating more or less its peritoneal tunic. They rise from the surface of the liver with a more gradual and uniform swell than the Tubera Circumscripta, and are, in different subjects, of various figures, sizes, colors, and consistence, often pulpy. No texture seems to escape the ravages of this fungus. It appears indifferently in all the viscera, in the cellular membrane, and even in the bones.

"Symptoms.—These vary in proportion to the varied seats of the disease: the diagnosis, therefore, must depend on one of the circumstances from which its name is derived, viz. its dispersion through many textures of the body. But, when this disease affects the liver in particular, then the symptoms will not materially vary from those which accompany the Tubera Circumscripta."

Two fatal cases of this disease, with the appearances on dissection, and a plate, complete the present fasciculus. From the author's experience, neither of the complaints above described are benefited by the use of mercury; "for, by the time that the most careful examiner can distinguish them, the progress of the disease has been already so considerable, that the mercurial action tends only to exhaust powers, which art will subsequently in vain attempt to restore." Although we cannot dispute the accuracy of this assertion, so humiliating to the powers of medicine, we venture to hope that a period may arrive, when the cause, origin, and progress, of these complaints shall be better understood; we even think that Dr. Farre's individual exertions will effect some improvement in our knowledge of them; and that his successful cultivation of the anatomy of morbid parts, will eventually produce a better result than the melancholy satisfaction of ascertaining the incurable nature of the disease.

---

*An Account of the Ravages committed in Ceylon by the Small-pox, previously to the introduction of Vaccination; with the Statement of the circumstances attending the introduction, progress, and success, of Vaccine Inoculation in that Island. By* Thomas Christie, M.D. 8vo. Cheltenham, 1811. pp. 104.

(Continued from p. 78.)

During the various attempts, recited in our last, for retarding the progress of the casual small-pox, the report of the Jennerian discovery reached the eastern world; and soon after the original works of Dr. Jenner, and that of Mr. Aikin, gave authenticity to the report. As early as the year 1800, attempts were made, but unsuccessfully, to transmit the vaccine fluid to India. Various other attempts, with a similar result, marked the endeavor to extend this blessing; but the difficulties were at length overcome.

"Several packets of matter were received at Ceylon from England by various surgeons; all of which were tried, but the length of the voyage had rendered it effete, and we were equally unsuccessful in our researches after the vaccine disease on cows, or the true grease in horses, which formed a principal object of inquiry in a tour round the island, in which I accompanied the governor, in the early part of 1802.

"The precaution had also been taken of writing to the medical men at Bombay and Madras, respecting the mode of forwarding the virus to Ceylon, in the event of the disease being produced at either of these presidencies; and Mr. North, with his usual anxiety for any improvement likely to contribute to the welfare of the people under his government, or the cause of humanity, had proposed that vessels, with proper subjects, should be sent to Bombay and the Persian Gulph, for the purpose of importing the virus on inoculated patients, if Lord Elgin should be fortunate enough to succeed in transmitting it from Constantinople.

"At length we had the pleasure to learn, that the object of our wishes was accomplished, and that the disease was produced at Bombay, and soon afterwards at Trincomalie, whence it was diffused over Ceylon, transmitted to the Coromandel Coast, and thence by Dr. Anderson, in the ship Hunter, to Bengal; but, as it seems of importance to trace distinctly the source of the matter which is so extensively diffused in the eastern world, and to account for its rapid extension throughout Ceylon, it will be satisfactory to copy some letters and other documents on this subject, which were written at the time, and most of them published in the different Indian papers.*

"At the period of the first introduction of the vaccine virus into Ceylon, a regular establishment existed for the promotion of small-pox inoculation, to which the natives were in a great degree familiarized, and it was only necessary to transfer the services of the individuals composing this establishment, to the preservation and diffusion of cow-pox, which the natives, in the first instance, seemed to consider as a milder species of the same disease; and it is to the decisive measure of government, in immediately adopting this plan, and the steady and liberal support afforded to it, that we ought to attribute the more rapid extension of the practice on its first introduction into Ceylon, than in Bengal, and some other parts of India, where, though similar measures were adopted, yet the establishment remained to be organized, and the prejudices of the natives against inoculation in general, at least as received from the hands of Europeans, to be overcome."

These

---

* It may not be unacceptable to our readers, to see the following account of the introduction and propagation of vaccination in India.

"*To the Editor of the Bombay Courier.*

"SIR, *Bombay, July* 2, 1802.

"For the satisfaction of the public, and the information of professional men in India, we beg of you to publish the following account of the introduction of cow-pox into this place. We have it now in our power to communicate the benefit of this important discovery to every part of India, perhaps to China, and the whole eastern world. We shall spare no pains in accomplishing a purpose so desirable, an object by which one of the greatest evils that has afflicted humanity may be diminished in a great degree, or even extinguished altogether.

"In the course of the last twelve months, we have repeatedly received by sea from England the vaccine matter, and many children have been inoculated to no purpose. We were not more successful with matter which was sent us directly by land from Constantinople.

Fortunately,

These unsuccessful experiments to introduce vaccination into the island of Ceylon, were followed, in August 1802, with one favorable case at Trincomalie, from which was supplied the material for spreading the practice to other parts.

---

Fortunately, Dr. Short, a surgeon of this establishment, residing at Bagdad, produced the disease at that place.

"He immediately forwarded the matter to Bussorah, where Mr. Milne, the surgeon of that presidency, also succeeded in infecting a patient with it. Mr. Milne soon afterwards inoculated a number of other children, and he sent the vaccine matter to Bombay by several ships. Even with this matter we were for a time unsuccessful, and after thirty or forty trials by various methods and by different surgeons.

"A fortunate inoculation at length produced the vaccine disease in Anna Dusthill, who is perhaps the first human being who underwent it in India. This child, the daughter of a servant of Capt. Hardie's, is about three years of age. She is very healthy, and certainly never had the small-pox. It is necessary to mention these circumstances, as from her alone the whole of the matter that is about to be sent all over India was first derived.

"We have received no history of the patients from whom it was taken at Bagdad and Bussorah, but we rest with confidence, from the knowledge of the medical gentlemen at those places, that no pains have been spared to make it pass through unexceptionable bodies.

"From Anna Dusthill, on the eighth day of her disease, and 22d of last month, seven children were inoculated; five of those, who certainly never had the small-pox, took the infection, and have already gone through nearly the whole course of the vaccine disease. The other two were not infected, but there is some probability that one of them has had the small-pox. From the five children that were infected, about thirty more have been inoculated, and a great many of them no doubt will take the disease. From these last we shall send the vaccine matter to the other presidencies, to Surat, Poonah, &c. &c.; and care shall be taken that none shall be employed but from an unexceptionable source.

"The vaccine disease in Anna Dusthill passed, as we have said, through its ordinary course, as described by writers on the subject.

"The pustule began to show itself about the third day; during the course of the fifth and sixth, she had slight symptoms of fever, and some uneasiness in the arm-pit of the inoculated side. The pustule on the eighth day was of the proper size for that period. It was flat, rather concave, and it consisted of many cells, which on being pricked gave out a transparent fluid. By the tenth day the inflamed areola round the pustule was extensive, and very distinct, in spite of the blackness of her skin. She had only a single pustule on the inoculated part, nor during the whole time did she suffer any material inconveniency from the complaint. All the five children who were inoculated from her, had a similar train of complaints. On two, whose parents parts. Of this event Dr. Christie gives an account in a letter to the Secretary to the Medical Board, at Bombay, dated Columbo, September 1, 1812, in which he says,

"That,

---

parents were Europeans, the inflamed areola, from the whiteness of the skin, was much more distinct than it had been on Anna Dusthill.

"We have thus detailed the progress of the symptoms, and we have no doubt but that this is the genuine cow-pox. Some surgeons here, who have seen the disease in England, are of the same opinion; we hope, therefore, that this will tend to quiet the apprehensions of parents, which in some instances we find to be very great; and that our experience at this place, so far as it has gone, will give confidence to practitioners.

"Almost all the medical men at this presidency have witnessed the disease, many of them are inoculating for it, nor do I understand that any difference of opinion has arisen concerning its nature.

"One test indeed we still want of its genuine nature, and that is, its power of preventing the variolous infection; to this test it shall shortly be put. As this island does not contain less than 150,000 people, sufficient supplies of children must arise to keep up the disease, even without any dependence on Salsette, or the neighbouring continent.

"The Hindoos and Persees here both shew the utmost desire of having their children inoculated with the vaccine disease. We shall instruct the native practitioners of physic regarding it, but on this part of the subject we are not without apprehensions. Whoever is sufficiently acquainted with what has been done in Europe, with regard to the cow-pox, is aware that some foreign poison, such as that of the small-pox, is apt to be mixed with it, whence a compound disease arises, or some other poisonous matter may be, from various causes, introduced, instead of the vaccine virus, whence a disease altogether different is produced. The History of the Count De Moffet, as detailed by Dr. De Carro, affords a most instructive lesson on this subject. The greatest care, therefore, should be employed, to warn the native practitioners, that the vaccine matter may be degraded, by many causes, and their utmost attention is necessary to prevent it.

"We can affirm from our own knowledge, that this government have anxiously assisted our wishes for procuring the vaccine disease, by the way of Bussorah. They represented to Lord Elgin the importance of it to this great society of mankind, and they called for the aid of the residents of Bussorah and Bagdad. Dr. De Carro, of Vienna, who has distinguished himself so honorably in this career, transmitted in the first instance the vaccine matter to Lord Elgin, who several times before had shewn us his attention to the subject. By his lordship's orders, it was sent to our resident at Bagdad, and again to the resident at Bussorah. To both those gentlemen the public are under great obligation, for the interest they took in the subject. Finally, it fell into the hands of Dr. Short and Mr. Milne, as we have already said, nor could it have been more fortunately placed.

"We

" That, after various failures at the different stations on this island, the vaccine inoculation has at length happily succeeded at Trincomalie, with matter taken from the arm of a child of European parents, at Bombay, on the eighth day from inoculation, being the 10th of July. The matter was sent by Dr. Helenus Scott, under cover to Mr. North, and did not reach Trincomalie till the 11th of August. Several patients were on that day inoculated with this matter, by Mr. Rogers, medical superintendant at Trincomalie; but, as in former cases, it failed of success in all, except in John Sybille, a half-cast boy, of twelve years of age, in whom it very fortunately took effect. On the 20th of August, being the ninth day from inoculation, a distinct pustule was formed on the inoculated part, bearing the characteristic marks of the cow-pox, and attended with pain in the axilla, and slight fever, so that Mr. Gilbert Hall, surgeon of the Malay corps, who, in consequence of the absence of Mr. Rogers, had charge of the patient, had no hesitation in pronouncing it to be the true vaccine disease. He immediately inoculated thirteen patients from the arm of the boy, in all of whom, he informs me, the inoculation had evidently taken effect, on the 26th of August. The most effectual measures have been ordered by this government for keeping up the disease, and extending it as expeditiously as possible to the different stations on this island, and to the Coromandel Coast, by means of inoculated patients. The fortunate event of John Sybille's inoculation with *matter thirty-two days old*, should teach us not to despair of success with dried matter, which has even been kept a considerable time in a hot climate; but the very frequent failures with dried matter, both in Europe and in India, shew that it is at best but a very uncertain means of conveying the contagion to any considerable distance."

After the fortunate event of the vaccination of John Sybille at Trincomalie, the vaccine disease was speedily communicated by vaccinated patients, or by impregnated threads, to every part of Ceylon. The history of the progress of vaccination in this island, its successes, its failures, and the opposition made to it, are detailed in, and authenticated by, various public documents, for the particulars of which our readers must be referred to Dr. Christie's pamphlet.

In 1803, there was considerable evidence of the security afforded against small-pox, by vaccination; but some in-

---

" We have been more particular than necessary for the medical profession, who must be supposed to be in possession of every fact that has occurred on the subject in Europe; but, as we shall disperse the vaccine disease very widely, and as it will affect, and as we hope it will promote the happiness of, every family, we wish to satisfy the public at large, concerning the sources from which we have derived it, and the foundation of our belief that it is of a genuine kind.

(Signed) " WILLIAM MOIR,
" H. SCOTT."

stances stances of failure having occurred, or being reported to have occurred, the necessary investigation was made to ascertain the facts, which did not prove permanently injurious to the reputation of vaccination.

In 1804, Dr. Christie made the tour of Ceylon, for the first time after the commencement of the Candian war, and found that the vaccine disease existed in perfect purity at all the principal stations in the island, and that the inhabitants generally placed confidence in the preservative efficacy of the cow-pox, except in the district of Jaffnapatnam, where the disease had become extinct, and the practice of vaccination got into disrepute, in consequence of a spurious disease having been introduced; after passing through which, several persons caught the small-pox and died.

"In 1805, the practice of vaccination continued to be followed with the most perfect success; and, although the small-pox prevailed in the Candian country in the month of May of that year, it did not extend to the British possessions; but, on the contrary, the alarm created by it on the frontier, increased the number of applications for vaccination; and it appears, from the report of May 1805, that the number of patients vaccinated during that month, in the Columbo district alone, amounted to 486; and that, on the south-west side of the island, we enjoyed a perfect exemption from small-pox.

"The impression which the security from small-pox acquired by the practice of vaccination, made on the minds of all classes of inhabitants in Ceylon, will be best expressed by extracts from the addresses presented to our most excellent governor, on his departure from the island, in July 1806.

"*Extract of an Address from the Civil, Judicial, and Military Officers, resident at Columbo, to his Excellency the Honorable Frederic North, Governor.*

"The natives under your government will long remember your Excellency with reverence and gratitude, as the founder of seminaries for their improvement in religion and knowledge, and of various institutions of charity for the relief of their sick and poor, and most particularly for the incalculable blessing you have brought on this island, by the successful introduction and rapid extension of vaccination."

"*Extract from the humble Address of the Native Headmen, &c. to his Excellency the Honorable Frederic North, &c. &c.*

"Your Excellency's care for their well-being extended itself so far as not only to favor them with lands for cultivation, but also, perceiving that the small-pox made every year a sad slaughter amongst the natives, (who from nature are fearful of the same in the highest degree,) did not spare any expense to accustom them to inoculate, by which means, at present that fatal complaint being extirpated, the old men and women in the interior of the country declare that they are become happy parents, through the preservation of their children by inoculation, [*vaccination*."]

"In

"In the year 1806, the number of vaccinations considerably increased; and in January of that year the contagion of small-pox became extinct in the district of Jaffnapatnam, by means of vaccination, which had been very successfully and extensively practised there since its re-establishment in March 1805.

"In February 1806, the only part of Ceylon where small-pox existed was Aripo, where a great concourse of strangers was assembled from the different parts of India for the purpose of attending the pearl fishery, and where the disease was introduced by a man who landed from the coast with small-pox, which unfortunately was communicated to a few other persons, before the necessary measures for prevention could be taken.

"On the breaking up of the fishery, in April, the disease was brought to Columbo, where we had enjoyed an exemption from it since 1804; but it did not spread to any extent, and the alarm occasioned by its appearance greatly increased the number of our vaccinations, at the same time that the existence of the contagion afforded us an opportunity of proving by variolous inoculation and exposure of our patients the continuance of the purity of our vaccine matter, and, as far as possible in the island, the permanency of its preservative efficacy, in opposition to the idea of Mr. Goldson, whose cases begun at this time to be talked of in Ceylon."

"A few cases of small-pox continued to occur at Columbo for some time, but no patient who had been vaccinated caught it; and the vaccinations, which were greatly increased by the circumstance, amounted in all to 54,958 at the end of the year 1806."

In 1807 the small-pox appeared and spread in the district of Trincomalie; in consequence of which Governor Maitland issued an advertisement, the success of which is evidenced by the report on the progress of vaccination published at the end of that year.

The progress of vaccination in 1808 and 1809, is detailed in the following letters:

"*To the Editor of the Ceylon Gov. Gazette.*

"I inclose an abstract of the number of patients vaccinated throughout the different districts in Ceylon during 1808, amounting to 26,207, which exceeds the proportion of any former year; and, when added to 76,828, the number previously vaccinated, makes a total of 103,035 persons who have been officially reported to me, as having regularly passed through the vaccine disease, since its first introduction into this island in 1802.

"The dreadful ravages which the small-pox usually committed in Ceylon, previously to the introduction of vaccination, must be in the recollection of every one; and it affords me infinite pleasure to observe, that, agreeably to the most certain information I have been enabled to procure, that destructive malady has not existed in any part of the British possessions on this island, during the year 1808, except in the district of Galle, into which it was brought on the 31st of January by a Maldivian boat last from Bengal.

"A large proportion of the crew of this boat died, and the disease was communicated by a fisherman, who visited it on its first arrival, to two or three of the inhabitants of the neighbourhood of Galle, but spread no further, which must be attributed chiefly to the favorable influence of vaccination, which had been so extensively diffused in that and the other districts of the island.

(Signed) "T. CHRISTIE.

"*Columbo,* 10*th Feb.* 1809."

"I subjoin, for more general information, an abstract of the number of patients vaccinated in the different districts in Ceylon, during 1809, amounting to 25,6[illegible]7; which, added to 103,035, the number vaccinated in former years, makes a total of 128,732 persons who have been officially reported to me by the respective superintendents and vaccinators, as having regularly passed through the vaccine disease, since its first introduction into this island in 1802, besides a few others inoculated by individuals, not belonging to the vaccination establishment.

"Agreeably to the best information I have been able to obtain, the small-pox has not existed in any part of this island since February 1808, till October last, when the disease was brought to Jaffnapatnam by a country boat from Quilon on the Malabar coast. The contagion spread to a few individuals, who had not been vaccinated in the Pettah of Jaffnapatnam, and, by means of a civil prisoner, was introduced into the jail of that place, but its progress was immediately arrested by the indiscriminate vaccination of all the other prisoners.

"By a late report from Mr. Stutzer, superintendent of vaccination at Jaffnapatnam, it appears that there were only six individuals ill of the small pox in that district, and it has found its way to no other part of the island except Putlam, where a Coolie from Jaffna was taken ill with small-pox in December last, but has since recovered without communicating the disorder to any other person.

"The vaccine disease has now been so extensively diffused throughout this island, that, while inoculations continue so frequent as at present, we can have no reason to apprehend that the contagion of small-pox will ever spread epidemically, in any part of the British possessions in Ceylon; and its occasional appearance here, has the good effect of proving the preservative efficacy of the vaccine, and of rousing the natives from their apathy on the subject, as exemplified at Jaffnapatnam, where 1830 people have been inoculated during the two last months, and amongst them several Bramins, men and women, who had hitherto declined submitting to the operation.

"I shall only add, that, with a view of proving the permanency of the preservative efficacy of cow-pox, and the continuance of the purity of the virus on this island, Mr. Stutzer has, at my request, in November and December last, inoculated with small-pox matter several patients who had passed through the vaccine disease in 1804 and 1809, all of whom have resisted the contagion.

(Signed) "THOMAS CHRISTIE."

"*Columbo,* 24*th Jan.* 1810."

To this account of the progress and success of vaccination in the island of Ceylon, to the final expulsion of the variolous lous contagion, the author subjoins some observations on the laws by which the vaccine disease appears to be governed in a tropical climate.

"According to the most attentive observation, the vaccine disease in India seems to have been governed by the same laws as in Europe, and the ordinary course of the disease, as remarked in Ceylon, agrees perfectly with the description given by the Medical Board of Bombay, in their letter, page 23.

"The vaccine matter had been constantly kept up in Ceylon, by a succession of patients, since its first introduction in the case of John Sybille, in August 1802, till the time of my leaving the island in February 1810, during which it had passed through at least 390 persons in succession; but its genuine nature had not been in the smallest degree altered, and its preservative efficacy remained the same, as proved by experiments at Jaffnapatnam, in November 1809; and, when properly applied to a person who had not had the cow-pox or small-pox, and was free from eruptions, and otherwise healthy, it seldom failed to produce the true vaccine vesicle.

"Considerable inflammation and ulceration were however occasionally excited in Ceylon from the insertion of vaccine virus; but these were of rare occurrence, when the vaccinators observed the directions given, to take it on the eighth day, or earlier.

"The conical turbid pustule was more frequently met with when the matter was taken at a late period, when a bad lancet was used, or when psora, or other herpetic eruptions, existed on any part of the skin; but in these cases the inoculation was always repeated, and very generally with success.

"Diseases of the skin, and particularly psora, are so frequent with the natives of Ceylon, that, had we objected to the inoculation of such cases, we should have excluded a great proportion of the mass of the people from the benefits of vaccination. We therefore made it a rule to give all such patients a trial, and by perseverance in repeated inoculations, choosing a clean part of the skin, and cautioning them against scratching or irritating the vesicle, often succeeded.

"It has already been shewn, that the most malignant disease of the skin which appears in Ceylon, or perhaps in any country, the Arabian leprosy, or elephantiasis of the ancients, of which there is a good description and drawing in Dr. Adams's 'Treatise on Morbid Poisons,' does not, in every case at least, render the constitution unsusceptible of either small-pox or the vaccine disease. It ought, therefore, it is conceived, to be laid down as a rule, that, in cases of epidemic small-pox, no other disease of the skin ought to prevent us from giving the patient a chance of protection from small-pox, by vaccine inoculation.*

---

* "It may here be mentioned, that I have successfully vaccinated in Ceylon two or three Albinos, born of Indian parents, in whom the areola, from the want of coloring matter in the skin, appeared as in Europeans; though this deviation from nature must be considered as a lusus, not a disease."

"The

"The more slight deviations from the regular vesicle, as described by Dr. Jenner, Dr. Willan, and others, were also occasionally observed in Ceylon; and, although these in general appeared to give security, and render the constitution unsusceptible of small-pox, or the vaccine disease in its regular form, the inoculations were in all such cases frequently repeated. A son of my own, who had a vesicle, regular in form, but premature, the areola having commenced on the seventh day, has been since inoculated six times without effect.

"The prevalence of small-pox, at the time of the introduction of the vaccine, afforded us frequent opportunities of seeing the occurrence of small-pox pustules in patients under vaccination, when the diseases were modified, or suspended by each other; but in this respect we observed no deviation from the appearances which have been so often and so well described, as occurring under similar circumstances in Europe.

"We have also met with cases in which the eruption of measles occurred on patients who had been vaccinated, and both diseases seemed to run their course at the same time, only that the progress of the vaccine areola was occasionally retarded for some days.

"Chicken-pox is of frequent occurrence in Ceylon, and often mistaken for small-pox; but I never chanced to meet with a case where it occurred in a vaccinated patient.

"Scarlatina is not known in Ceylon, nor agreeably to my information in any part of India, where contagious typhus seldom or never occurs, so that we have had no opportunity of observing the effect of these diseases, in modifying the action of the vaccine virus.

"The effect of some chronic eruptions, in sometimes rendering the constitution unsusceptible of the vaccine disease, or occasioning spurious pustules, has already been mentioned; and it is only necessary to add, that, during the seven years in which the cow-pox was so extensively diffused in Ceylon, I never saw one case of eruptive disease, which could with propriety be attributed to the effects of vaccination.

"Diseases of the skin, particularly the various species of psora,* herpes, and tinea, were remarkably frequent in Ceylon before the introduction of cow-pox, and are so still, but certainly they have not been increased in frequency by that cause; on the contrary, I have occasionally seen slight cutaneous affections removed by vaccination.

"In the letter of Mr. Lodovice, a case is mentioned where the disease was communicated from one human subject to another by casual contact, a circumstance which occurred three or four times in Ceylon; and which is not to be wondered at when the habits of the

* "I have occasionally, in Ceylon, met with a species of psora, in which the live insect could be picked out with a needle or pin, and seen with the naked eye. I have not had an opportunity of examining it with a microscope, but conceive it must be the same as that described by Dr. Adams as occurring at Madeira.—*See Adams on Morbid Poison.*"

people people are attended to, in wearing very little clothing, and crowding together in the night, whole families often sleeping on the same mat.

"The circumstance of the disease being communicated by casual contact, induced me to try if it could be produced without any wound, the idea of which alarmed many of the natives; but all attempts to communicate it in this way, by applying vaccine matter to a sound part of the skin, and retaining it there by pledgits and sticking-plaister, failed of success.

"It was remarked, that, during the rainy season, we more frequently failed in communicating the disease, which might in some degree be owing to the greater frequency of catarrh at that season, but was also attributed to the matter being diluted, or washed away by the rain, few of the natives of the lower order wearing any covering on their arms, on which account I used to caution them against exposing their arms to wet, or bathing on the day of the inoculation.

"Secondary pustules were not an unfrequent occurrence, on the first introduction of the vaccine disease, when small pox was prevalent in the island, and were probably in some instances dependant on variolous effluvia, but they also now and then appeared, though more rarely, after the expulsion of small-pox; and from experiment we ascertained, that the true vaccine vesicle might be produced by matter from the secondary pustule."

